# Factors for viral infection in blood donors of South Kivu in the Democratic Republic of Congo

**DOI:** 10.11604/pamj.2014.19.385.4328

**Published:** 2014-12-17

**Authors:** Jeff Maotela Kabinda, Dramaix-Wilmet Michèle, Philippe Donnen, Serge Ahuka Miyanga, Jef Van den Ende

**Affiliations:** 1 Provincial Blood Transfusion Centre of Bukavu. Democratic Republic of Congo; 2 Catholic University of Bukavu, Democratic Republic of Congo; 3 Research Centre for Biostatistics, Epidemiology and Clinical Research, Brussels, Belgium; 4 Free University of Brussels; Brussels, Belgium; 5 School of Public Health, Brussels, Belgium; 6 Research Centre for Health Policy and Systems / International Health; 7 Provincial General Referral Hospital in Bukavu; 8 Institute of Tropical Medicine, Clinical Sciences, Antwerp, Belgium

**Keywords:** Blood donation, transfusion, HIV prevalence, hepatitis B, hepatitis C

## Abstract

**Introduction:**

Assessing the knowledge, attitudes, practices and behaviors among blood donors in South Kivu and identify risk factors for viral markers.

**Methods:**

A descriptive and analytical cross-sectional study involved 595 blood donors in the city of Bukavu (Head city of the province of South Kivu) in the eastern Democratic Republic of Congo.

**Results:**

Our sample consisted of 70.3% men with a median age of 23 and 77% of young people fewer than 30 years. The score of knowledge and attitude of blood donor's volunteer on blood safety were assessed at 23.5% and 79.1%. A statistically significant difference was observed between the loyal and new blood donors volunteer (25.1% vs 64.6% p < 0.001); between blood donors volunteer of low and high education level (p = 0.04). Motivation to donate blood in 95.9% of cases respect ethical rules of donation. The prevalence of viral markers in blood donors is as follows: 4.8% hepatitis B, 3.9% hepatitis C, 1.6% HIV. For HIV, the low level of education and replacement blood donors are most at risk, the antigen of hepatitis B is observed in blood donors over 30 years, blood donors living couple.

**Conclusion:**

General knowledge on blood safety is very low in the first link in the chain transfusion (blood donors). A good education of this population conducted by the transfusion service reinforced building (training and support) is needed.

## Introduction

In sub-Saharan Africa there is a high incidence of several diseases and pathological conditions that complicate directly or indirectly by anemia [1–4]. Democratic Republic of Congo (DRC.) is not immune to these diseases malaria, hemoglobinopathies, obstetric haemorrhage and nutritional deficiencies. To correct the often severe anemia, in most cases, a blood transfusion is indicated [3, 4]. Unfortunately at the same time, African countries and the Democratic Republic of Congo. in particular, are facing highly endemic communicable diseases by blood and inadequate organization of blood transfusion services, increasing the risk of contamination of recipients through blood products [3, 5]. Thus the prevalence of viral and parasitic markers is generally high. It varies between 0.6% and 16% for the human immunodeficiency virus (HIV), 5 to 25% for the surface antigen of hepatitis B ( HBsAg) and 0.5% to 3% for hepatitis C [3]. In Democratic Republic of Congo, prevalence differs depending on location, in Kinshasa between 2001 and 2004 HIV in blood donors ranged from 5.94% to 6.1% and HBsAg between 3.63 and 9.2% [6], syphilis was 1.05% and the hepatitis C virus (HCV) was 4.3% between 2002 and 2004 [7] and in North- Eastern country in 2007 HIV was 4.7%, syphilis 3.7% and 5.4% HBsAg [8]. Transfusion infection risk can be significantly reduced by some measures involving the accountability of donor blood candidate [9]. Among these measures the self-exclusion of the donor candidate blood before his risky behavior and clinical selection before donation [4, 10–13] by health workers. This selection is a step in the general clinical examination done before the donation. It aims to search medical cons -indications to donate blood [10, 14]. Results of these strategies are remarkable in industrialized countries [4]. The reasons given by Nedié this success would be a high level of education and knowledge of people in these countries [4]. In the Democratic Republic of Congo as Africa, the level of education of the population is not as high, an information to the public is essential. The present study aims to make an inventory by assessing of knowledge, attitudes and practices of blood donation and transfusion in blood donors of South Kivu and to identify risk factors for viral infection in the blood donors.

## Methods

### Type and scope of the study

It is a descriptive and analytical cross-sectional survey. It focused on blood donors who attended the hospital blood banks of the general provincial referral hospital in Bukavu (2000 donations per year on average), the general referral hospital Panzi (800 donations per year on average) and the general hospital Katana (500 donations per year on average) between 1 January 2011 and 30 June 2011. The provincial and the Panzi hospital are teaching hospitals and are in the health district of Bukavu, the capital of the province of South Kivu while the hospital is in the Katana health zone with the same name 50 km north of Bukavu in the province of South Kivu in the eastern Democratic Republic of Congo. These general hospitals, 34 in number in the province, are allowed to perform activities of blood donation, collection of blood bags in place of the Provincial Blood Transfusion Center (PBTC) Bukavu who has not yet operational capacity to serve the entire province of blood in quantity and quality. The PBTC is one of 11 provincial centers in the country which are headed by the National Blood Transfusion Center (NBTC) in Kinshasa. In the province, blood donations are made by blood donors belonging to non-profit blood donors’ volunteer associations. There are thirty associations scattered throughout the province and grouped into two platforms: association of voluntary blood donors and the federation of voluntary blood donors. Voluntary blood donors of these platforms receive training from PBTC or non-governmental organizations.

### Study population and material

The sample size was calculated by the software OpenEpi by introducing a 50% factor (knowledge) results in a population, a confidence level of 95% and a precision of 5%. On this basis minimum 384 subjects were included. We recruited 595 blood donors including 408 at the provincial hospital, 70 and 117 at Katana and Panzi. The inclusion of blood donors in each hospital structure was made randomly and has been to recruit the (volunteer, paid and family) donor between 18 and 65 years of age with an odd number in the order of arrival. The interviewers were nurses or laboratory technicians attached to the blood bank of the health institution and trained in blood safety by BPTC. In these health centers, pre-donation interview with using a questionnaire was carried out before each donation to detect cons -indications to donate blood. This interview was conducted either by a doctor or by a nurse responsible for transfusion in the institution trained for this purpose. For blood donors included in the study, an additional questionnaire was proposed. The questionnaire contained closed-ended questions and was pre-tested. The questions focused on the general characteristics of blood donor motivation to donate blood, sexual behavior, knowledge about research communicable diseases (HIV window, concept of risk), the consequences of blood transfusion and personal history. Before administering the questionnaire an explanation has been provided by the nurse and an informed consent was signed. For the donors included in this study and that who were allowed to donate blood after the pre-donation interview, the blood sample was obtained at the time of collection of the blood bag. For donors included in the study but temporarily or permanently prohibited donation, the blood sample was taken after obtaining their verbal consent. This sample was used to determine HIV, HCV, HBV prevalence's using the same methods as those made for any biological qualification of blood donation at the blood bank. We used Determine HIV1 - 2TM Determine HBsAg and HCV Determine tests.

### Variables

We selected as variables for analysis: age ( 18-30 years, 31-40 years, 41 years and older), sex, marital status, without, primary, secondary level of education (and higher) but we dichotomized ( low and high) in the statistical analysis. Low for primary school and those who did not attend school at all, high for those who attended secondary and tertiary levels of education. Blood donors were divided into volunteers, family and paid; the last two categories were grouped into the category of replacement donors. The sexual behavior of the donor, knowledge of the consequences of transfusion, the different attitudes of blood donor and different knowledge of blood donor were taken into account. “Motivation to donate blood ” variable was divided into two parts: the motivation to donate blood for transfusion safety (I gave blood to save lives, because the media talk about, as peers, because I was transfused and no explanation) and motivation to donate blood not for blood safety (I gave blood for the detection of diseases, to be paid because a parent is hospitalized and because of the pressures of the environment). In the overall assessment of the knowledge of volunteer blood donor on blood transfusion we used a score considering the 9 categories of knowledge and we found a very good knowledge among blood donors who had a score of 7 correct answers and a good knowledge for those who were 5-6 and a lack of knowledge for a score less than 5. For general evaluation of positive attitudes towards blood safety we have achieved a score taking into account the correct answers expected from all sections of the attitude variable except for the responses to the question “during the interview is what that donors do they give truthful answers? The attitude was measured using a score: a score of ≥4 was considered a very good attitude, a score of 3 meant that the blood donor had a good attitude and a score of ≤ 2 reflected a bad attitude in volunteer blood donors. In the analysis, the “knowledge” and “attitude” variables were dichotomized to avoid small sample size.

### Statistical analysis

After data collection, entry thereof is organized using the software EPI Info Version 3.5.1. Analyzes were made by the same software and also by STATA version10. The descriptive analysis was performed through calculations proportions for discrete or categorical variables, the median, minimum and maximum for age. The test Pearson chi-square or Fisher exact (when necessary) was used to compare proportions; the significance level used was 0.05. Analyzes of knowledge and attitude were made only among volunteer blood donors trained in transfusion safety, while analyze for the seroprevalence of virus were made on the whole sample. In fact we are currently doing an assessment of the knowledge and attitude on volunteer blood donors who have received training. The association between independent and dependent variables (prevalence of HIV, HCV and HBsAg) was judged by the odds ratio (OR) with a 95% confidence interval (95% CI).

## Results

### Demographics of blood donors in South Kivu

Sociodemographic characteristics of the study population, as shown in Table 1 below, are as follows: 593 subjects, 70.3% were men; the median of age of blood donors is 23 years with a minimum of 18 and a maximum of 64 years. More than three quarters of the blood donors were under 30 years, 71% were single, 60% had secondary education and 5% are illiterate, 88.9% are volunteer donors which 78.7% loyal.

**Table 1:** General characteristics of blood donors in Bukavu, eastern Democratic Republic of Congo

	n	%
**Sex**	593	
Female	176	29.7
Male	417	70.3
**Age (years)**	585	
18-30	449	76.8
31-40	85	14.5
41 and above	51	8.7
**Marital status**	587	
In couple	170	29.0
Single	417	71.0
**Level of education**	590	
Primary	48	8.1
Secondary	354	60.0
Higher	160	27.1
Without	28	4.7
**Blood donation category**	577	
Volunteer	513	88.9
Familial	60	10.4
Paid	4	0.7
**Volunteer blood donor category**	497	
New blood donor	106	21.3
Former blood donor	391	78.7

### Knowledge and attitudes of volunteer blood donors

Motivation to donate blood in 95.9% of cases in accordance with the rules of ethics of blood donation, among them 93.5% said they gave their blood to save lives. Outside of volunteering among 513 blood donors volunteer, we observed that four blood donors came for screening, a person gave blood hoping for a cash consideration and 16 persons (3.1%) have given a parent who was hospitalized. Related to sexual behavior, almost all blood donors volunteer (96.5%) attended at most one sexual partner in the last 6 months. Four percent of blood donors volunteer had sex against money. Our respondents stated at 85.9%, we could trust the answers given by the volunteer's blood donors during interviews pre- donations. Face exposure to a risk of infection, volunteers blood donors behave differently, 18.1% expect the appointment of the next blood donation while 81.9% blood donors volunteer make a gift of blood to know their HIV status after exposure. The proportion of responses on the concept of the window is low (21%). This low percentage is also evident in the knowledge of the consequences of blood transfusion (39.8%). The proportion of donors who know their HIV status for hepatitis B and hepatitis C are low (31.0% and 28.3% respectively) ( Table 2).

**Table 2:** Knowledge and attitudes of volunteer blood donors in South Kivu, eastern Democratic Republic of Congo

	Respondents	%*
**Knowledge**		
Screening for HIV during blood donation	511	91,0
Screening for hepatitis during blood donation	511	38,0
Screening for syphilis during blood donation	508	34,4
Screening for malaria during blood donation	510	36,1
HIV Serological window	445	20,9
HIV serological status before blood donation	511	77,9
HBV serological status before blood donation	510	31.0
HCV serological status before blood donation	509	28.3
Transfusion consequences	513	39.8
**Attitudes**		
Transfusion security as a motivation for blood donation	511	95.9
Number of sexual partners during the last 6 months	486	
0 – 1	469	96.5
≥ 2	17	3.5
Behaviour facing a contamination risk exposure	470	
Waiting for the next blood donation appointment	85	18.1
Immediate blood donation in order to know the serological status	385	81.9
Having the results of serological tests immediately after blood donation	512	94.3
Blood donors true answer during blood pre-donation interview	512	85.9
Sexual intercourse for money	505	4.4

* Percentage of correct responses expected

**Figure 1:**
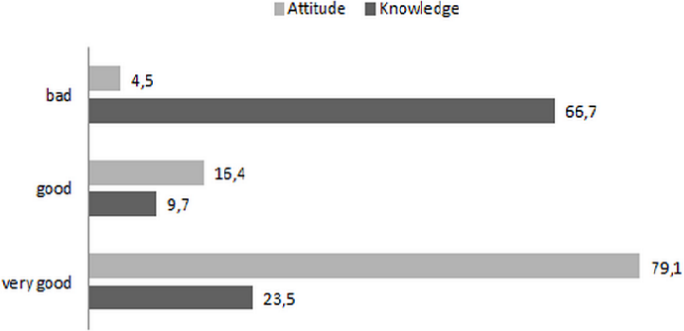
Assessment of proportion of knowledge and attitude on blood safety volunteer blood donors in the Democratic Republic of Congo

Overall rating of knowledge of blood donors volunteer shows that 23.5% of blood donors volunteer have a satisfactory knowledge on blood safety while the proportion of blood donors with a good attitude is 79.1% as shown in Figure 1. This general knowledge differs significantly between loyal blood donors volunteer and new (25.1% vs 64.6% p < 0.001) and voluntary donors of low level and high level of education (p = 0.04). New blood donors are more informed than older donors and, low level of study donors are better informed than those highly educated. The proportion of subjects with a good knowledge does not differ significantly by gender (p = 0.24), age (p = 0.94) and marital status (p = 0.30) ( Table 3). Specifically this knowledge is poor in topics such as research on blood donation hepatitis, syphilis and malaria with percents respectively 38.0%, 34.4% and 36.1%. Good attitude on blood safety is different depending on the characteristics except for sex as we see in Table 3.

**Table 3:** Knowledge and attitude of blood donors volunteer based on general characteristics

	Knowledge			Attitude		
	n	Good(%)	p	n	Good(%)	p
**Age (years)**			0,94			0.02
< 30	336	33.0		340	81.8	
> 30	97	33.3		99	70.7	
**Sex**			0,24			0.94
Female	130	29.2		133	78.9	
Male	312	34.9		313	79.2	
**Marital Status**			0,30			<0.001
In couple	122	36.9		121	83.3	
Single	318	31.8		324	67.8	
**Level of education**			0,04			<0.001
Low	38	47.4		40	35.0	
High	402	31.6		404	83.4	
**Donation Loyalty**			<0,001			<0.001
New donor	79	64.6		73	63.0	
Former donor	351	25.1		361	83.7	

This table shows the association between the general characteristics of blood donors and the knowledge and attitude

### Viral risk factors among volunteer blood donors

The result of the prevalence of viral markers in blood donors shows that the prevalence of hepatitis B was 4.8%. Antibodies against the hepatitis C were found in 3.9% of cases and HIV was found in 1.6% of blood donors. As shown in Table 4, we did not observe significant differences in seroprevalence of HIV and hepatitis C by knowledge and attitude of blood donors volunteer but the proportion of HBsAg was statistically higher in voluntary donors who had a good knowledge compared to those who did not have( 7.1% vs 1.4%) p = 0.007. The distribution of HIV prevalence shows that there is no statistically significant difference according to age, sex, marital status and the category of voluntary blood donor. HIV prevalence differed significantly among blood donors low level of education and those with higher education levels (5.5% vs 1.0% p = 0.04), blood donors and replacement volunteers (6.6% vs 1.0% p = 0.001). Blood donors of low level of education are more affected by HIV than those with a high level of study OR = 5.5 (95% CI 1.1 to 26.3). Among blood donors replacement, HIV prevalence is higher than in voluntary donors OR = 6.8 (95% CI 1.9 to 24.2). For the surface antigen of hepatitis B, blood donors over 30 years are more affected than those with less than 30 years. In blood donors living in couple, the proportion of HBsAg is higher than in singles and in blood donors replacements prevalence was statistically higher than in volunteer donors ( Table 5) while no difference in prevalence of antibodies against hepatitis C based on these general characteristics.

**Table 4:** The prevalence of infectious markers depending on the knowledge and attitude of blood donors volunteer

	HIV				HBsAg				HCV			
	n	%	OR (95% CI)	p	n	%	OR (95% CI)	p	n	%	OR (95% CI)	p
**Knowledge**				0.51*				0.007				0.23
Bad	282	0.4	0.2 (0.0-2.8)		282	1.4	0.2 (0.0-0.6)		277	5.8	1.9 (0.6-5.9)	
Good	141	1.4	1		141	7.1	1		131	3.1	1	
**Attitude**				0.99*				0.11*				0.99*
Bad	91	1.1	1.9(0.2-20.6)		91	6.6	2.9(0.9-8.5)		90	4.4	0.8(0.3-2.7)	
Good	335	0.6	1		335	2.4	1		322	5.0	1	

* Fisher exact. OR:odd ration.CI: confidence interval

**Table 5:** Socio-demographic factors of viral infectious risk among blood donors in Bukavu

	HIV				HBsAg				HCV			
	n	%	OR (95% CI)	p	n	%	OR (95% CI)	p	n	%	OR (95% CI)	p
**Total**	568	1.6			568	4.8			544	3.9		
**Age (years)**				0.47				<0.001				0.30
<30	429	1.3	0.6 (0.16-2.2)		429	3.0	0.3 (0.1-0.6)		407	3.4	0.6 (0.2-1.5)	
>30	130	2.3	1		130	10.0	1		128	3.9	1	
**Sex**				0.64				0.09				0.96
Female	165	1.2	0.6 (0.0-2.9)		165	2.4	0.4 (0.14-1.15)		158	3.8	1.0 (0.4-2.5)	
Male	402	1.7	1		402	5.7	1		385	3.9	1	
**Marital Status**				0.06				0.02				0.66
In couple	159	3.1	1		159	8.2	1		156	4.5	1	
Single	402	1.0	0.3 (0.1-1.0)		402	3.5	0.4 (0.19-0.87)		381	3.7	0.8 (0.3-1.9)	
**Level of education**				0.04*				0.09*				0.42*
Low	73	5.5	5.5 (1.1-26.3)		73	9.6	2.5 (1.0-6.14)		71	1.4	0.3 (0.0-2.4)	
High	492	1.0	1		492	4.1	1		470	4.3	1	
**Blood donation category**				0.001				0.001				0.10
Replacement donation	61	6.6	6.8 (1.9-24.2)		61	13.1	3.7 (1.6-8.8)		57	0.0	0 (0.0-1.5)	
Volunteer donation	491	1.0	1		491	3.9	1		472	4.4	1	
**Volunteer donor category**				0.99*				0.33*				0.15*
New donor	102	1.0	0.9 (0.1-8.2)		102	5.9	1.8 (0.7-5.1)		91	1.1	0.2 (0.0-1.6)	
Former donor	373	1.1	1		373	3.2	1		365	4.9	1	

## Discussion

### Knowledge and attitude on blood donation and blood transfusion

This exploratory study was aimed to assess the knowledge of blood donors on blood donation and transfusion. Our sample was composed of a majority of young people (76% under 30 years), blood donors of secondary education level and males. A significant proportion of young male donors was found in other African studies [2, 4, 6, 8, 15, 16] and reflects the youth population of the African people and blood donors. The number of female subjects is limited because of physiological reasons, women give less blood regularly [15]. The high percentage of blood donors in secondary education level is explained by the fact that the blood donor associations are composed of students attending secondary schools and are predominant in the population within the age of 18 and 65, required to donate blood.

Knowledge of blood donors volunteer on certain aspects of blood safety was low as in Burkina Faso [4]. But the odds of correct responses are elevated in blood donors who had low level of education and among new donors. In the Democratic Republic of Congo, as part of the response against HIV, sensitization and training blood donors volunteer, especially those responsible for blood donor associations are among the pillars of blood safety activities. This lack of knowledge among former donors may have two explanations firstly a problem with training and a second difficulty of assimilation in blood donors. While the attitude score is higher in the group of former volunteer donors. The desire was to have in this group; repeatedly trained and aware; a high score in knowledge and attitudes. This contradiction results leads us to ask: is that it is a satisfaction or routine in the group of former donors or other identifying factor that explains this? In this case, the thinking should be conducted to determine the responsibility of blood donors volunteer [9] and the trainers. Given the differences between the score of the attitudes and knowledge; theoretically we would like both scores go together, we believe that our volunteer blood donors apply to adopt best learned attitudes that are consistent with safe blood without a control of the basics of communicable diseases by blood. One possible reason that could explain this is that these attitudes are common to the prevention of other diseases transmitted by blood or sex. But our study cannot provide information on this point; this will be covered in a qualitative study that should be conducted in voluntary donors to understand the difference between knowledge score and attitude. Evaluations of training provided should be made to identify ways of improving the quality of training and knowledge of blood donors. These assessments should be systematic because these formations blood donors are carried out by health workers trapped in the logic paradigm Pasteur [17] characterized by a very hierarchical health system where priority is given to the technical aspects, a planning top to bottom, and maximalist normative, leaving little room for care providers and the opinion of the population ( blood donors). Add to this disease perdiemite or financial snacks distributed during training, awareness and blood collection. This disease is observed in health workers or the heads of associations and even among blood donors at the expense of the quality of work [17].

Africa is characterized by attitudes of solidarity and the primary motivation to donate blood for a family member [4]. This is contrary to the results found in this study but is consistent with the data from 4 African countries described by Tagny et al [15]. In our environment there is a dynamic grouping of blood donors associations that began before the existence of the organization of blood transfusion services by the health authorities. These members of civil society associations are non-profit and organize promotional activities in favor of voluntary blood donation. These actions maintain the voluntary blood donation in the big cities but this reality is not the same in rural areas where health family donation is predominant because of cultural beliefs and lack of information [15]. However there is a problem when 4 out of 5 volunteers face exposure to risk donors, arrange to donate blood in order to be screened. This situation can be attributed to strong awareness campaigns in the community, in the media and in schools conducted by the program against AIDS / HIV. This has installed a public confusion between blood donation and screening [4].

The good results of the clinical pre-donation selection is based on several factors: the relationship during the servicing between the blood donor and health workers, the acceptable level of knowledge of the donor on donation and transfusion communicable diseases by blood and competence of health workers [4, 11, 18, 19]. This interview should bring a candidate donor blood to self- exclude. However, our study shows that only 21% of donors knew the window period and less than 40% had sufficient knowledge of the research of hepatitis, syphilis and malaria in each unit of blood collected. Doctor- donor relationship can be measured by the degree of accuracy of the information collected during the interview, even though our study did not reach this level of evidence, however, indicates that 14% of information provided by donors volunteers are false. This rate is lower than the results in Burkina Faso where it was 34% [4]. Nedié and al advocated the need to establish a relationship of trust, as taught by any human medicine, however there is always a possibility of under-reporting [4, 20], given the complexity of human and discouragement of African doctor before some paradigms [17] and to the socio -economic situation dilapidated. The other problem is that this interview is not always done by qualified personnel and on time, it is often done by nurses in an environment of mobile blood collection without adequate and favorable conditions.

### The risk factors for viral markers in blood donors

Democratic Republic of Congo as other sub-Saharan countries have not yet reached the detection of viral markers of HIV and hepatitis on 100% of pockets transfused [3]. Nevertheless, an effort was made to a systematic screening of these three viruses is done. The supply disruptions reagents are the cause of this low screening rate. These supplies are, in most cases, possible thanks to funding from international organizations. The result of the seroprevalence found joined the results found in the Democratic Republic of Congo and other African countries [3, 4, 6–8, 16, 21, 22]. Comparing our results and those of the study conducted in the same environment as ours, we note that the seroprevalence of these viruses instead of diminishing increases. Thus Namululi [22] found HIV seroprevalence 1% between 2001-2005 and HBsAg 3.7% against 1.6% for HIV and 4.8% for HBsAg in our study. Moreover, the study Namululi et al showed that the gift of family blood had decreased from 14.1% to 5.1% from 2001 to 2005. This decrease was attributed to the recruitment model of blood donor (strong clinical selection). In our study the proportion of family donors is 10.4%, an increase of 51% between 2005 and 2011. This difference in proportion of family donation between the two studies can be explained by the fact that Namululi have worked with donors attending one institution while we targeted three institutions. In addition, between 2001 and 2005 there was an influx of funding (World Bank, Belgian cooperation...) which supported the associations of blood donors. Will it finance this activity to infinity in order to hope to maintain an acceptable percentage of voluntary donations. These blood donors are accustomed and attracted snacks distributed after each blood donation and distributed elements in the promotional activities of blood donation. All these actions have a cost and are supported by various funds that have an end. It would be better to make blood donors and volunteers really responsible. But the process may not have an impact in the context of poverty. One solution would be to propose that blood donors support in giving impetus by funding from revenue-generating activities. Dahourou and all [23] propose a series of mechanisms that could prevent blood donations replacement and increase voluntary blood donation and reduce the risk of infection. These measures include the revitalization of associations of blood donors, the identification of new sites of mobile blood collection and intensification of mobile blood drives. These measures require largely financial support to the transfusion service.

Namululi had found in their study that the risk was lower for a volunteer donor, male, old donor. Our results confirm that HIV, voluntary blood donors and those with a high level of education are at lower risk. For hepatitis B: blood donors less than 30 years, single and volunteers were at lower risk. Blood donors replacement and low level studies have a high risk of infection as revealed by other African series cited earlier in this work. Indeed, in this group there is a chance to have some curious and poor people, secretly paid by family and concealing information during the selection interview [21, 24, 25]. Donors over 30 years old are also at risk of infection, this age is a period of intensive sexual activity favorable to the transmission of different viruses in the population who share the same mode of transmission. Concerning the investigation, several studies have shown that it was not a guarantee of safe blood for these studies found an association between a high level of education and knowledge and attitudes contrary to blood safety, as do the gift for screening and non adequate knowledge of the window [4, 26]. In our sample, the number in the group of low level of study is low which might not be able to detect the difference in the two groups. In this study singles are at lower risk than those living with a partner, this is may be so by chance. The profile of a blood donor at a lower risk of infection varies depending on the studies and it will be difficult to find a perfect profile [27]. Attention should be paid on the whole of transfusion chain to reduce risk of infection by implementing quality assurance measures appropriate to the context.

## Conclusion

Reduction of transfusion risk starts with thorough control of the first element in the transfusion chain that is to say, the blood donor. This study has shown that knowledge on blood safety and blood borne diseases among blood donors is very low; the prevalence of viral markers increases and the family donation becomes more important. Capacity building at all levels is imperative. It will be for the transfusion service supported by opinion leaders to conduct an education of the population. But we should think about hiring a technician in sociology and communication in the transfusion service to improve the quality of awareness and training of medical and paramedical staff involved in the transfusion chain. A focus should be placed on the control of pre-donation selection.
